# Thymus-Derived, Peripherally Derived, and *in vitro*-Induced T Regulatory Cells

**DOI:** 10.3389/fimmu.2014.00017

**Published:** 2014-01-24

**Authors:** Eyad Elkord

**Affiliations:** ^1^United Arab Emirates University, Al Ain, UAE; ^2^University of Salford, Manchester, UK; ^3^University of Manchester, Manchester, UK

**Keywords:** thymus-derived, peripherally-derived, induced Tregs, cancer, autoimmune diseases, GvHD, infection

T regulatory cells (Tregs) are key players in immune regulation of both physiological and pathophysiological conditions. There are different Treg subsets but they can be divided into two major subsets: natural Tregs (nTregs) and adaptive/induced Tregs (iTregs); or as recently named, thymic-derived Tregs (tTregs) and peripheral-induced Tregs (pTregs), respectively. In addition, there are two subsets (Tr1 and Th3) of FOXP3− iTregs. It is my pleasure to introduce to our scientific community this timely research topic, bringing 21 contributions from several groups. These articles shed more light on the origin, differentiation, phenotype, specificity, function, and role of the different Treg subsets in different disease settings.

The first article by Sakaguchi’s group elegantly discusses the recent progress of the epigenetic modifications associated with the functional stability of Tregs (1). FOXP3 on its own is not sufficient for conferring developmental and functional characteristics of Tregs, and simultaneous induction of Treg-specific epigenetic changes and FOXP3 expression are required for lineage specification and functional stability of Tregs. Future studies should focus on understanding the molecular pathways of both epigenetic changes and FOXP3 expression to identify ways for generation and expansion of stable Tregs for therapeutic approaches.

The next five articles improve our understanding of the different Treg subsets. Povoleri et al. provides a comprehensive review of the molecular signatures and induction processes, mechanisms of action, lineage stability, and differentiating characteristics of both thymus and peripheral FOXP3+ and FOXP3− Tregs (2). While there are two main Treg subpopulations, a great deal of lineage plasticity exists. Therefore, understanding mechanisms of Treg induction, suppressive function, and lineage stability is vital for unraveling the role of different Treg subsets in human diseases. Currently, Treg-based therapy is considered as a feasible approach to treat human diseases, however, the optimal use of Tregs in therapy relies on our further understanding of Treg plasticity as well as their epigenetic/miRNA profiling. The next article nicely reviews the phenotypic and functional differences between tTregs and pTreg subsets, and discusses the difficulty in distinguishing these subsets (3). While FOXP3 is a key marker for Treg development and function, its sole expression is not useful to discriminate between activated T cells, bona fide Tregs, or even between different Treg subsets and additional markers are required. The validity and controversy of some of the recently identified markers, including Helios, LAP/GARP, and Neuropilin-1, as markers of tTregs and activated Tregs, are discussed. The review by Goldstein et al. addresses the role of three important cytokines including IL-2, TGF-β, and TNF-α in differentiation and homeostasis of tTregs and pTregs (4). TNF-α inhibitors indicate that part of their anti-inflammatory effect could be mediated by their action on Tregs; however, limited information is available and more work is required to understand the effect of TNF-α on Tregs. Cytokine administration or blocking are in many clinical trials to modulate inflammatory diseases, therefore a better understanding of cytokine effects on the induction and/or expansion of Treg subsets should provide insights on improving the efficacy of immunotherapeutic modalities. The following review focuses on iTregs, while making comparisons to nTregs, and their function and approaches to induce their generation *in vivo* and *in vitro* as a promising therapeutic target (5). It is clear that more markers remain to be elucidated to accurately define iTregs. Human autoimmune diseases are characterized by a reduction in Treg numbers and/or function, and iTregs might have the potential to restore tolerance to treat autoimmune diseases. The molecular mechanisms of inducing the generation of iTregs, both *in vivo* and *in vitro* are discussed. It is concluded that a complex of regulated series of interactions with FOXP3 are required for establishing Treg stability. The following review focuses on both dendritic cells (DCs) and Tregs and the role of DCs in controlling antigen-specific nTregs and iTregs in the periphery (6). The authors give details on how different subsets of DCs play different roles in induction and expansion of nTregs and iTregs. There are specialized DC subsets in peripheral locations that act to expand nTregs or to induce the generation of FOXP3+ iTregs from CD4+ FOXP3− T cells.

Role and function of Tregs in cancer is a major focus in this research topic due to the important role that these cells play in dysregulation of anti-tumor immunity. The next five articles review our current knowledge and give us more insights on this important topic. Adeegbe and Nishikawa comprehensively focus on the involvement of nTregs in various animal models and human tumors (7). They further discuss iTregs and the relationship and cooperation with nTregs to dampen immune responses against tumors. They provide evidences supporting the role of nTregs in cancer with less consensus on the role of iTregs because of the lack of their precise definition. Further understanding of the function of both iTregs and nTregs and their discrimination in each tumor setting will certainly help future therapeutic approaches to eliminate or block these cells for improving anti-tumor immunity and clinical benefits. The next comprehensive review covers the current agreements and discrepancies on the role of tTregs and pTregs in cancer (8). Mechanisms of Treg expansion in tumors remain controversial because both tTreg proliferation and iTreg generation may happen in the same tumor setting. The authors propose innovative immunotherapeutic strategies to divert unstable/uncommitted Treg, mostly enriched in the pTreg pool, into tumor-specific effector cells, while preserving systemic immune tolerance mediated by self-specific tTreg. Treg levels are not only increased in the blood of cancer patients, but they are also significantly elevated within tumor tissues; therefore the focus of the next review is on Tregs in the tumor microenvironment (9). It is vital to understand the processes of Treg elevation in cancer patients and to identify the specific mechanisms involved in their accumulation within the tumor. These mechanisms could include chemokine-mediated recruitment of FOXP3+ Tregs, induction of Tregs, and proliferation of tTregs within the tumor microenvironment. Additionally, potential strategies for targeting the different mechanisms of Treg enrichment in tumor microenvironment in attempts to improve cancer immunotherapy are discussed. Wainwright et al. reviews Tregs in brain cancer, providing details of their phenotype, mechanisms involved in their pathogenesis, and therapeutic strategies to target these cells in brain tumor (10). The features of brain tumors determine the nature of tumor-infiltrating Tregs. In this particular cancer setting, the authors propose that tTregs are the key players contributing to tumor progression and failure of immunotherapies. Indoleamine 2,3-dioxygenase 1 (IDO) is overexpressed in brain tumor and its critical involvement in regulating the levels of tumor-infiltrating Tregs is a major focus of this article. The next review discusses the role of Tregs in cancer development with a focus on early events following the interactions between tumor and the immune system (11). Number and quality of Tregs recruited to the tumor microenvironment in the very early stage have a significant impact on the outcome of anti-tumor immunity and subsequent tumor development. The authors propose that pTregs are unlikely to have much impact in most cancers because the fate of the tumor is being decided early, and preventive vaccines against cancer should be considered while avoiding therapeutic vaccines, as they could worsen host tolerance to tumor antigens.

The next three articles focus on different disease settings. Beres and Drobyski elegantly review the role of Tregs in the biology of graft versus host disease (GVHD) (12). There is a persistent reduction in peripheral Treg levels of patients with high clinical grades of acute GVHD, compared to patients with lower grade acute GVHD or no GVHD. Although there has been a significant understanding of the role of Tregs in GVHD, it remains unclear about the exact role of each Treg subset (e.g., tTregs, pTregs, CD8+ Tregs) and further studies are required. Exploiting FOXP3+ Tregs provides a promising approach to treat GVHD in patients. Preclinical data and clinical studies using Tregs as an adoptive cellular therapy for the prevention of GVHD in human are presented. Bluestone’s group presents their opinion in this hypotheses and theory article regarding the role of pTregs in immune homeostasis and autoimmunity (13). Some cell surface markers and transcription factors, such as Neuropilin-1 and Helios, which may distinguish tTreg from pTreg subsets *in vivo* are discussed. It is proposed that pTregs have a distinct phenotype and function from tTregs and *in vitro* generated Tregs. While tTregs are central to immune homeostasis and prevention of autoimmunity, pTregs have specialized functions depending on the type of inflammation, and they have vital roles in certain settings such as mucosal immunity and fetal tolerance. The next review discusses the signals that activate tTregs once entering peripheral lymphoid tissues (14). The authors provide evidence, mainly from their own work, and propose that tTregs can, upon activation in the presence of antigen, become antigen-specific Tregs with stronger suppressive capacity; this is dependent on late Th1 and Th2 cytokines, and not the early cytokines IL-2 and IL-4.

The next three articles focus on the role of Tregs in infection. The first article details the role of CD4+ FOXP3+ Treg subsets in HIV infection (15). Treg quantification and function in HIV infection remain controversial because of the lack of specific Treg markers to identify the different human Treg subsets, in addition to the discrepancies originated from different approaches to analyze Tregs. For a better interpretation of the role of Tregs in HIV, both percentages and absolute Treg numbers, in addition to the stage of HIV infection should be considered. The recent findings of the existence of phenotypically and functionally distinct human CD4+ FOXP3+ Treg subsets may provide more insights on understanding the effect of Tregs on HIV and effect of HIV on Tregs. In the next research article, Germanidis et al. examined liver biopsies from patients with chronic hepatitis B virus (HBV) for the expression of different immunosuppression-related genes (16). They report that the immunosuppressive environment of liver is down-regulated on maintained long-term remission in comparison with active disease. The following review summarizes the different Treg subsets and their function in filarial parasite infection (17). Although, it is agreed that chronic filarial infection is associated with increases of most of the Treg subsets; IL-10-mediated regulation by Tr1 cells, along with conventional IL-10-producing Th2 cells, is the most consistent finding. Defining precise markers for the different Treg subsets should provide more insights into understanding their role and mechanisms of action and as potential therapeutic targets in many disease setting including parasitic infections.

The last group of these series is categorized as four miscellaneous articles. Due to its pleotropic actions and great significance in immunomodulation, Wraith’s group describes in detail, the regulation of the adaptive immune responses by IL-10 (18). This review focuses on IL-10 produced by FOXP3+ tTregs and pTregs, FOXP3− pTregs, and different T helper subsets. Our better understanding of the role of IL-10 in immunomodulation gave the opportunity to design more efficient, antigen-specific immunotherapies for clinical applications including allergic and autoimmune diseases. While IL-10 and TGF-β are the most commonly studied immunosuppressive cytokines, the recently identified IL-35 has been shown to have potent suppressive functions *in vitro* and *in vivo*. In this regard, Olson et al. review the structure and function of IL-35 as a key mediator of suppression of T effector cells with the potential to propagate infectious tolerance through the generating of potent IL-35-secreting inducible Tregs (iTr35) (19). The next review focuses on different iTreg-mediated immunosuppressive mechanisms, specifically adenosine (ADO) and prostaglandin E2 (PGE2), which can compromise anti-tumor immune responses (20). The authors propose the significance of ADO- and PGE2-mediated suppression in cancer patients. Pharmacologic interventions designed to selectively target ADO and PGE2 pathways could not only inhibit the tumor-derived factors but also silence the suppressive activities of Tregs and thus restore the anti-tumor activity of T effector cells. The last research article shows that a subpopulation of CD25hiTNFR2+ cells generated *in vitro* from CD4+ cells through TCR stimulation express FOXP3 and other Treg markers, but have effector functions rather than suppressive characteristics (21).

In summary, a considerable progress has been made in understanding the role and function of Treg subsets in different disease settings. Further understandings of the molecular pathways and their mechanisms of action and defining surface markers specific for the different Treg subsets should provide chances to use Tregs in the clinic for treating different diseases or to target them to enhance anti-tumor/microbial immune responses.
